# FISST Based Method for Multi-Target Tracking in the Image Plane of Optical Sensors

**DOI:** 10.3390/s120302920

**Published:** 2012-03-02

**Authors:** Yang Xu, Hui Xu, Wei An, Dan Xu

**Affiliations:** School of Electronic Science and Engineering, National University of Defense Technology, Changsha 410073, China; E-Mails: simon863@vip.sina.com (H.X.); nudtanwei@tom.com (W.A.); stfan79@yahoo.com.cn (D.X.)

**Keywords:** finite set statistics, signal amplitude information, PHD filter, multi-target tracking, Gaussian mixture

## Abstract

A finite set statistics (FISST)-based method is proposed for multi-target tracking in the image plane of optical sensors. The method involves using signal amplitude information in probability hypothesis density (PHD) filter which is derived from FISST to improve multi-target tracking performance. The amplitude of signals generated by the optical sensor is modeled first, from which the amplitude likelihood ratio between target and clutter is derived. An alternative approach is adopted for the situations where the signal noise ratio (SNR) of target is unknown. Then the PHD recursion equations incorporated with signal information are derived and the Gaussian mixture (GM) implementation of this filter is given. Simulation results demonstrate that the proposed method achieves significantly better performance than the generic PHD filter. Moreover, our method has much lower computational complexity in the scenario with high SNR and dense clutter.

## 1. Introduction

Optical sensors have been widely applied both in important military and civil areas due to their properties of long detection range, high concealment ability and large coverage area. Since there is usually a long distance between targets and sensor which generates a low SNR and dense clutter scenario, multi-target tracking in the image plane of optical sensors is a very difficult problem. In multi-target tracking, the aim is to estimate the number of a set of targets and the state of each target from a set of measurements received. However, due to the variation of targets number with time in the field of view of the sensors and the existence of miss detection and dense clutters, multi-target tracking in image plane remains a challenging problem.

In addition to the location measurement, amplitude information has been proven to improve tracking performance [1–5]. One of the pioneering techniques was the approach proposed by Colegrove, Lerro and Bar-Shalom [1,2] where the probability data association (PDA) filter utilizing target amplitude was applied in the context of single-target tracking. The target amplitude has also been incorporated in the multiple hypothesis tracking (MHT) framework [3] and Viterbi data association scheme [4]. More recently, the significance of target amplitude has been explored for data association of closely spaced targets in [5]. Although significant progress has been made recently, the approaches mentioned above are based on data association technique, which requires expensive computational cost in most circumstances. Therefore, the traditional data association approach is not the optimal option for multi-target tracking in image plane of optical sensor in the scenarios with time-varying targets number, low SNR and dense clutters.

A good candidate is the emerging Bayesian approach in the framework of finite set statistics (FISST) proposed by Mahler [6]. FISST provides a set of mathematical tools that allows direct application of Bayesian inferencing to multi-target problems. PHD proposed by Mahler [7] is a computation tractable approximation for the optimal multi-object Bayes filter based on Finite Set Statistics (FISST). Operating on the single-target state space, the PHD filter avoids the combinatorial problem that arises from data association and thus leads to superior performance in comparison with traditional MHT algorithm in multi-target tracking [8,9]. These features render the PHD filter extremely attractive to many researchers. The primary PHD applications can be found in [10–13]. In [14], Clark *et al*. incorporated target amplitude into a PHD filter for the first time in multi-target tracking, which improves the tracking performance. However, their amplitude is modeled for radar and sonar application and the computational complexity analysis of their method is absent.

In this paper, we propose a FISST based method which uses signal amplitude information in a PHD filter for multi-target tracking in the image plane of optical sensors. Based on analyzing the imaging characteristics of the optical sensor, we model the signal amplitude and incorporate it into the PHD in the form of amplitude likelihood ratio. In the situation where the SNR of target is unknown, we then present an alternative method based on this amplitude model. Simulation results demonstrate a significant improvement of the proposed method in tracking performance. Furthermore, the computational complexity of our method in the scenarios with different clutter density and SNR is also discussed.

This paper is organized as follows: Section 2 models the signal amplitude generated by the optical sensor. Section 3 demonstrates how to incorporate the amplitude information (for known and unknown SNR case) into a PHD filter and then the GM implementation for this filter is given. Section 4 presents the simulation results that validate the proposed method. The conclusions are given in Section 5.

## 2. Amplitude Measurement Model

### 2.1. Amplitude Likelihood Ratio

In an optical multi-target tracking system, the original images taken by the sensor are usually processed by background suppression before being further used for target tracking. Assuming the noise is addictive, we define the SNR *d* of the residual image as [15–17]:

(1) d=sσ

where *s* is the mean signal value of target, *σ* is the standard deviation of the residual image. Each measurement from the image consists of the two-dimensional position vector *z* in the image plane and the corresponding amplitude *a* ≥ 0, that is, a measurement vector has the form ***z̃***:=(***z****^T^*, *a*)*^T^*. For simplicity, we assume that the value of the target signal has no spreading in the image plane. We assume that the noise is Gaussian, then the probability densities of the amplitude of the false alarms and the target *p*_0_(*a*) and *p*_1_(*a*|*d*) can be written as [18]:

(2) $$p_{0}(a)=\frac{1}{\sqrt{2\pi \sigma^{2}}}\text{exp}\left(-\frac{a^{2}}{2\sigma^{2}}\right)$$

(3) $$\begin{array}{l} p_{1}(a|d)=\frac{1}{\sqrt{2\pi \sigma^{2}}}\text{exp}\left(-\frac{{\left(a-s\right)}^{2}}{2\sigma^{2}}\right)\\ \;\;\;\;\;\;\;\;\;\;\;\;\;\;=\frac{1}{\sqrt{2\pi \sigma^{2}}}\text{exp}\left(-\frac{{\left(a-\sigma \cdot d\right)}^{2}}{2\sigma^{2}}\right) \end{array}$$

This leads to the probabilities of false alarm and detection $p_{\mathrm{FA}}^{\tau }$ and $p_{D}^{\tau }(d)$ with a detection threshold *τ*,

(4) $$p_{\mathrm{FA}}^{\tau }=\int_{\tau }^{\infty }p_{0}(a)\mathrm{da}=\text{erf}(\tau /\sigma )$$

(5) $$p_{D}^{\tau }(d)=\int_{\tau }^{\infty }p_{1}(a|d)\mathrm{da}=\text{erf}(\tau /\sigma -d)$$

where $\text{erf}(x)=\frac{1}{\sqrt{2\pi }}\int_{x}^{+\infty }\text{exp}\left(-\frac{y^{2}}{2}\right)\mathrm{dy}$ is the probability error function. According to Equations (4) and (5), we have:

(6) $${\text{erf}}^{-1}\left(p_{\mathrm{FA}}^{\tau }\right)-{\text{erf}}^{-1}\left[p_{D}^{\tau }(d)\right]=d$$

In target tracking application we usually choose $p_{\mathrm{FA}}^{\tau }$ as a fixed value. Given $p_{\mathrm{FA}}^{\tau }$, the threshold *τ* can be calculated via the inverse form of Equation (4) with the parameter *σ*, and the probability of detection $p_{D}^{\tau }(d)$ can be calculated via Equations (5) or (6) for the target with SNR = *d*. Table 1 lists the values of $p_{D}^{\tau }(d)$ for different SNR values *d* and specified $p_{\mathrm{FA}}^{\tau }$ with *σ* setting to be a normalized value, *i.e.*, *σ* = 1.

When the target SNR *d* is known, we can get our amplitude likelihood functions for the false alarm and the target as:

(7) $$c_{a}^{\tau }\;(a)=\frac{1}{p_{\mathrm{FA}}^{\tau }}\;p_{0}\;(a),\;\;\;\;\;a\geq \tau$$

(8) $$g_{a}^{\tau }\;(a|d)=\frac{1}{p_{D}^{\tau }(d)}\;p_{1}(a|d),\;\;\;\;\;a\geq \tau$$

The amplitude likelihood ratio given a threshold *τ* is defined as [19]:

(9) $$\rho_{a}^{\tau }\;(a|d)=\frac{g_{a}^{\tau }\;(a|d)}{c_{a}^{\tau }\;(a)}$$

We use the notation *c_a_*(*a*) and *ρ_a_*(*a*|*d*) to denote the case where *τ* = 0, then we have:

(10) $$c_{a}\;(a)=p_{0}\;(a)$$

(11) $$\rho_{a}(a|d)=\frac{p_{1}(a|d)}{p_{0}(a)}$$

From Equations (5) and (9) we can see that the calculations of $p_{D}^{\tau }(d)$ and $\rho_{a}^{\tau }(a|d)$ rely on a specified known target SNR, however, this requirement cannot be satisfied in most practical tracking systems. We adopt an alternative approach to circumvent this issue next.

### 2.2. Method for Unknown SNR

When the SNR of target is unknown, one straightforward approach would be to estimate the unknown parameter *d* from the measurement amplitudes *a*. However, this approach requires a large number of measurements from the target to achieve an accurate estimate of *d*. Furthermore, due to the unknown association between measurements and targets in multi-target environment with clutter, the approaches of estimating *d* usually fail. Similar to the idea introduced in [14], we adopt an alternative approach where we do not attempt to estimate *d* at all. Instead we marginalize out the parameter *d* over the range of possible values and find a probability of detection for $p_{D}^{\tau }$ and a likelihood ratio for $\rho_{a}^{\tau }$ that is not conditional on *d*.

Consider that we always have some prior information about the targets being tracked, so we assume that *p*(*d*), defined on the possible SNR values [*d*_1_,*d*_2_], gives the expected probability distribution of SNR values. Since the amplitude distribution in Equations (2) and (3) are symmetric thus have no biases in high or low SNR targets, the reasonable choice for *p*(*d*) is the uniform distribution *U*[*d*_1_,*d*_2_]. Then we define the probability of detection and amplitude likelihood ratio where SNR is unknown as:

(12) $$p_{D}^{\tau }=\int_{d_{1}}^{d_{2}}p(\nu )p_{D}^{\tau }(\nu )d\nu$$

(13) $$\rho_{a}^{\tau }\;(a)=\int_{d_{1}}^{d_{2}}p(\nu )\rho_{a}^{\tau }\;(a|\nu )d\nu$$

From Equations (5) and (12) we have:

(14) $$p_{D}^{\tau }=\frac{1}{d_{2}-d_{1}}\int_{d_{1}}^{d_{2}}\text{erf}(\tau /\sigma -\nu )d\nu$$

Note that the $p_{D}^{\tau }$ over the marginalized region [*d*_1_, *d*_2_] can be computed with numerical integration offline since it does not need to be computed at each iteration. The computation of $\rho_{a}^{\tau }\;(a)$ as in Equation (13) can be simplified and will be presented in Section 3.2.

## 3. PHD Filter with Signal Amplitude Information

Suppose that at time *k* there are *N_k_* target states ***x***_*k*,1_, ⋯ ***x***_*k*,*N*_*k*__, each taking values in a state space X ⊆ R^*n*_*x*_^; and *M_k_* measurements (detections) ***z***_*k*,1_, ⋯ ***z***_*k*,*M*_*k*__, each taking values in the observation space Z ⊆ R^*n*_*z*_^. In PHD filter, a multi-object state and a multi-object observation are represented by RFS:

(15) $$\begin{array}{l} X_{k}=\left\{x_{k,1},\cdots x_{k,N_{k}}\right\}\in F\;(X)\\ Z_{k}=\left\{z_{k,1},\cdots z_{k,M_{k}}\right\}\in F\;(Z) \end{array}$$

where F(X) and F(Z) are the finite subsets of X and Z, respectively. The state ***x*** = (*x*,*y*,*ẋ*,*ẏ*)*^T^* of each target contains the position (*x*, *y*)*^T^* and velocity (*ẋ*,*ẏ*)*^T^* in the image plane, while the measurement ***z*** is defined in Section 2. We assume that each target follows a linear Gaussian dynamical model and the sensor has a linear Gaussian measurement model, *i.e.*,

(16) $$f_{k|k-1}\left(x|x^{\prime }\right)=N\;\left(x;\;F_{k-1}\;x^{\prime },\;Q_{k-1}\right)$$

(17) $$L_{z}\;(z|x)=N\;(z;\;H_{k}\;m,\;R_{k})$$

where N(.;***m***,***P***) denotes a Gaussian density with mean *m* and covariance ***P***, ***F***_*k*−1_ is the state transition matrix, ***Q***_*k*−1_ is the process noise covariance, ***H****_k_* is the observation matrix, and ***R****_k_* is the observation noise covariance.

### 3.2. The PHD Recursion with Amplitude Information

We abbreviate the PHD filter incorporated with amplitude information as AI-PHD filter. Next we derive the prediction and update equations of AI-PHD filter based on the amplitude likelihood ratio given by Section 2. For simplicity, we do not consider target spawning in this paper.

**Step 1. Prediction:** The prediction equation of AI-PHD filter is the same as generic PHD filter since their state vector and state transition matrix are the same, *i.e*.,

(18) $$D_{k|k-1}\;(x)=\gamma_{k}\;(x)+\int (p_{S,k}(x^{\prime })\;f_{k|k-1}\;(x|x^{\prime }))D_{k-1|k-1}\;(x^{\prime })dx^{\prime }$$

where *γ_k_*(***x***) is the birth term for new targets, *p_S,k_*(***x′***) is the probability of target survival, *f_k|k_*_−1_(***x***|***x′***) is the transition density and *D*_*k*−1|*k*−1_(***x′***) is the PHD at time *k* − 1.

**Step 2. Update:** The update equation is changed when incorporated with amplitude information. Analogized to the update equation of generic PHD filter in [7], we have the update equation of our AI-PHD filter as

(19) $$D_{k|k}(x)≅L_{{\tilde{Z}}_{k}}(x)\cdot D_{k|k-1}(x)$$

where *L*_*Z̃*_*k*__(***x***) is the pseudo-likelihood function as

(20) $$L_{{\tilde{Z}}_{k}}\;(x)=1-p_{D}\;(x)+p_{D}\;(x)\sum_{\tilde{z}\in {\tilde{Z}}_{k}}\frac{L_{\tilde{z}}(x)}{\lambda \cdot V\cdot c(\tilde{z})+D_{k|k-1}\left[p_{D}\;L_{\tilde{z}}\right]}$$

(21) $$D_{k|k-1}\;\left[p_{D}\;L_{\tilde{z}}\right]=\int \left\{p_{D}\;(x)L_{\tilde{z}}\;(x)D_{k|k-1}(x)\right\}dx$$

where *λ* and *V* are the clutter density and area of image plane of optical sensor respectively. Assuming the amplitude *a_k_* is independent with target state *x_k_*, we can rewrite *L*_*Z̃*_*k*__ (***x***) and *c*(***z̃***) as

(22) $$L_{\tilde{z}}\;(x)=L_{z}\;(x)g_{a}^{\tau }\;(a|d)$$

(23) $$c\;(\tilde{z})=c\;(z)c_{a}^{\tau }\;(a)$$

where *L_z_*(***x***) is the measurement location likelihood function and *c*(***z***) is the probability density of the false alarm spatial distribution in the image plane. We assume that the targets are within the surveillance region of sensor, the probability of detection for a given threshold *τ* is then only dependent on *d*

(24) $$p_{D}\;(x)=p_{D}^{\tau }\;(d)$$

Substituting Equations (5), (8) and (22–24) into Equation (20) we have the pseudo-likelihood function of AI-PHD as

(25) $$L_{{\tilde{Z}}_{k}}\;(x)=1-p_{D}^{\tau }\;(d)+p_{D}^{\tau }\;(d)\sum_{\tilde{z}\in {\tilde{Z}}_{k}}\frac{\rho_{a}^{\tau }\;(a|d)L_{z}\;(x)}{\lambda \cdot V\cdot c(z)+p_{D}^{\tau }\;(d)\cdot \rho_{a}^{\tau }\;(a|d)\cdot D_{k|k-1}\left[L_{z}\right]}$$

Equations (18) and (25) compose the recursion of AI-PHD filter. The probability of detection $p_{D}^{\tau }\;(d)$ and amplitude likelihood ratio $\rho_{a}^{\tau }\;(a|d)$ are replaced by $p_{D}^{\tau }$ and $\rho_{a}^{\tau }\;(a)$ respectively for the unknown SNR case.

We can simplify the computation of $\rho_{a}^{\tau }\;(a)$ by noting the fact that $\rho_{a}^{\tau }\;(a)$ is calculated combined with $p_{D}^{\tau }\;(d)$ in Equation (25). From Equations (8) and (9) we have

(26) $$p_{D}^{\tau }\;(d)\cdot \rho_{a}^{\tau }\;(a|d)=\frac{p_{1}(a|d)}{c_{a}^{\tau }\;(a)}$$

Hence, instead of computing $\rho_{a}^{\tau }\;(a)$ by Equation (13) we can compute the expression $p_{D}^{\tau }\cdot \rho_{a}^{\tau }\;(a)$ directly using the method introduced in Section 2.2, *i.e.*,

(27) $$p_{D}^{\tau }\cdot \rho_{a}^{\tau }\;(a)=\int_{d_{1}}^{d_{2}}p(\nu )\frac{p_{1}\;(a|\nu )}{c_{a}^{\tau }\;(a)}d\nu =\frac{1}{\sigma \cdot c_{a}^{\tau }\;(a)\cdot (d_{2}-d_{1})}\left[\Phi \left(d_{2}-\frac{a}{\sigma }\right)-\Phi \left(d_{1}-\frac{a}{\sigma }\right)\right]$$

where $\Phi (x)=\frac{1}{\sqrt{2\pi }}\int_{-\infty }^{x}\text{exp}\left(-\frac{t^{2}}{2}\right)\mathrm{dt}$ is the standard normal distribution function which can be computed easily. Consequently, our approach incorporates the amplitude information into PHD filter with only a minor additional computational load.

We show the consistency of our AI-PHD filter with the generic PHD filter. If the SNR of target is set as *d* = 0, from Equations (7–9) we have $\rho_{a}^{\tau }\;(a|d)\equiv 1$. This is the condition under which our AI-PHD filter degenerates to the generic PHD filter.

### 3.3. Gaussian Mixture Implementation

An analytic solution to the PHD filter can be found under linear assumptions on the system and observation equations with Gaussian process and observation noises as described in Equations (16) and (17) [20]. In this case, both the prediction and update equations of PHD are represented by a mixture of Gaussians where the means and covariances are updated with Kalman filter and the weights of the Gaussian components are found using the PHD filter equations. We use Gaussian Mixture implementation of our filter for its simplicity in calculation and convenience of target state extraction comparing to the Sequential Monte Carlo (SMC) method [21].

We assume that the survival probability is state independent, *i.e*., *p_S,k_*(***x′***) = *p_S,k_* and the detection probabilities are $p_{D}^{\tau }\;(d)$ and $p_{D}^{\tau }$ for the known SNR and the unknown SNR case respectively. The intensity of the target birth RFS are Gaussian mixture of the form

(28) $$\gamma_{k}\;(x)=\sum_{i=1}^{J_{\gamma ,k}}\omega_{\gamma ,k}^{\left(i\right)}\;N\;\left(x;m_{\gamma ,k}^{\left(i\right)},\;P_{\gamma ,k}^{\left(i\right)}\right)$$

where $J_{\gamma ,k}$, $\omega_{\gamma ,k}^{\left(i\right)}$, $m_{\gamma ,k}^{\left(i\right)}$, $P_{\gamma ,k}^{\left(i\right)}$, *i* = 1, ⋯ *J_γ,k_*, are given model parameters that determine the shape of the birth intensity.

We assume a uniform location distribution of clutter in the measurement space, so that the clutter location likelihood is not dependent on the state or the measurement. Hence the clutter location distribution is constant over the measurement space and equals to the reciprocal of the area of the image plane of optical sensor, *i.e*., *c*(***z***) = 1/*V*. Next we give the prediction and update equations of Gaussian mixture implementation of our AI-PHD filter.

**Prediction:** The posterior intensity at time *k* − 1 is a Gaussian mixture of the form

(29) $$D_{k-1|k-1}\;(x)=\sum_{i=1}^{J_{k-1}}\omega_{k-1}^{\left(i\right)}N\;\left(x;m_{k-1}^{\left(i\right)},P_{k-1}^{\left(i\right)}\right)$$

where *J*_*k*−1_ is the number of Gaussian terms with the weights $\omega_{k-1}^{\left(i\right)}$, means $m_{k-1}^{\left(i\right)}$ and covariances $P_{k-1}^{\left(i\right)}$. Then the prediction intensity is still a Gaussian mixture as

(30) $$D_{k|k-1}\;(x)=\gamma_{k}\;(x)+p_{S,k}\sum_{i=1}^{J_{k-1}}\omega_{k-1}^{\left(i\right)}N\;\left(x;m_{S,k|k-1}^{\left(i\right)},\;P_{S,k|k-1}^{\left(i\right)}\right)$$

where the birth intensity *γ_k_*(***x***) is given by Equation (28) and the means $m_{S,k|k-1}^{\left(i\right)}$ and covariances $P_{S,k|k-1}^{\left(i\right)}$ are computed with the Kalman filter prediction.

**Update:** We rewrite the predicted intensity *D*_*k*|*k*−1_(***x***) as a Gaussian mixture of the form

(31) $$D_{k|k-1}\;(x)=\sum_{i=1}^{J_{k|k-1}}\omega_{k|k-1}^{\left(i\right)}N\;\left(x;m_{k|k-1}^{\left(i\right)},\;P_{k|k-1}^{\left(i\right)}\right)$$

Substituting the Equations (31), (20–25) into Equation (19), we obtain the intensity of our AI-PHD filter updated by measurements set *Z̃_k_* as the Gaussian mixture form

(32) $$D_{k|k}\;(x)=\left[1-p_{D}^{\tau }\;(d)\right]D_{k|k-1}\;(x)+\sum_{i=1}^{J_{k|k-1}}\sum_{\tilde{z}\in {\bar{Z}}_{k}}\omega_{k}^{\left(i\right)}(\tilde{z})N\;\left(x;m_{k|k}^{\left(i\right)}(\tilde{z}),\;P_{k|k}^{\left(i\right)}\right)$$

where the updated means $m_{k|k}^{\left(i\right)}(\tilde{z})=m_{k|k}^{\left(i\right)}(z)$ and covariances $P_{k|k}^{\left(i\right)}$ are calculated with the Kalman filter update. The updated weights $\omega_{k}^{\left(i\right)}(\tilde{z})$ in Equation (32) are computed as

(33) $$\omega_{k}^{\left(i\right)}(\tilde{z})=\frac{p_{D}^{\tau }(d)\cdot \rho_{a}^{\tau }(a|d)\cdot \omega_{k|k-1}^{\left(i\right)}N\;\left(z;{\hat{z}}_{k|k-1}^{\left(i\right)},\;S_{k|k-1}^{\left(i\right)}\right)}{\lambda +p_{D}^{\tau }(d)\cdot \rho_{a}^{\tau }(a|d)\sum_{l=1}^{J_{k|k-1}}\omega_{k|k-1}^{\left(l\right)}N\left(z;{\hat{z}}_{k|k-1}^{\left(l\right)},\;S_{k|k-1}^{\left(l\right)}\right)}$$

where ${\hat{z}}_{k|k-1}^{\left(i\right)}=H_{k}\;m_{k|k-1}^{\left(i\right)}$ is the predicted measurement and $S_{k|k-1}^{\left(i\right)}=H_{k}\;P_{k|k-1}^{\left(i\right)}\;H_{k}^{T}+R_{k}$ is the innovation covariance.

In the PHD update equation, Equation (32), and the weight update Equation (33), we replace the probability of detection $p_{D}^{\tau }(d)$ and term $p_{D}^{\tau }(d)\cdot \rho_{a}^{\tau }(a|d)$ by $p_{D}^{\tau }$ in Equation (14) and $p_{D}^{\tau }\cdot \rho_{a}^{\tau }(a)$ in Equation (27) when the SNR is unknown.

## 4. Simulation

In this Section, by setting up multi-target tracking simulation in the image plane of optical sensor, we examine the performance and computational complexity of our method for known and unknown SNR cases and benchmark them with generic PHD filter with different combinations of probability of false alarm and SNR value.

### 4.1. Simulation Scene and OSPA Metric

**Figure 1.**
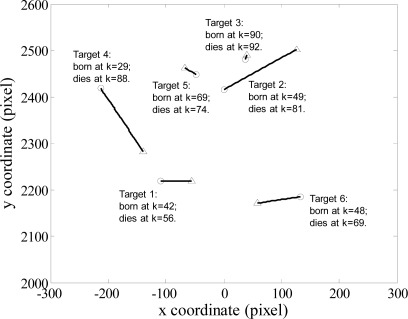
Target trajectories in the pixel plane with start/stop position as O/▵.

Consider a scenario with an unknown and time varying number of targets in clutter in the image region [−300, 300] × [2,000, 2,600] (*pixel*). Up to *N_k_* = 6 targets are generated in this region with the random birth and dieing time instants. Figure 1 shows the true trajectories of each target. All targets in each simulation had the same mean SNR (this is not necessary by the algorithm but simplifies the presentation of results).

Each target has survival probability *p_S,k_* = 0.99 and follows the linear Gaussian dynamics in Equation (16) with:

(34) $$F_{k-1}=\left[\begin{array}{cc} I_{2} & \Delta I_{2}\\ 0_{2} & I_{2} \end{array}\right],\;\;\;\;\;\;\;\;\;\;\;\;Q_{k-1}=\sigma_{\nu }^{2}\left[\begin{array}{cc} \frac{\Delta^{4}}{4}\;I_{2} & \frac{\Delta^{3}}{2}\;I_{2}\\ \frac{\Delta^{3}}{2}\;I_{2} & \Delta^{2}\;I_{2} \end{array}\right]$$

where Δ = 1 s is the sampling period, and σ*_v_* = 0.5(*pixel*/*s*^2^) is the standard deviation of process noise. The location measurement follows the observation model in Equation with $H_{k}=[I_{2}0_{2}],\;R_{k}=\sigma_{\varepsilon }^{2}I_{2}$, where σ*_ε_* = 1(*pixel*) is the standard deviation of measurement noise.

The intensities of the birth RFS are Gaussian mixtures of the form:

(35) $$\gamma_{k}\;(x)=\sum_{i=1}^{6}0.1N\;\left(x;m_{\gamma }^{\left(i\right)},\;P_{\gamma }^{\left(i\right)}\right)$$

where $m_{\gamma }^{\left(i\right)}$ is chosen around the mean initial states of i-th target, $P_{Y}^{\left(i\right)}=[50,50,15,15]$, i = 1,⋯6. To mitigate the exponential growth of mixture components, at each time step the number of Gaussian components is capped to a maximum of *J*_max_ = 100 components, whilst pruning is performed with a weight threshold of *T* = 10^−5^, and merging is performed with a threshold of *U* = 2.

We adopt the optimal subpattern assignment (OSPA) metric [22] for the purpose of multi-target performance evaluation since it jointly captures errors in the target state and target number estimates. Given two arbitrary finite sets *X* = {***x***_1_, ⋯ ***x****_m_*} and *Y* = {***y***_1_, ⋯ ***y****_n_*}, the OSPA is computed as follows:

(36) $${\bar{d}}_{p}^{\left(q\right)}\;(X,\;Y)=\{\begin{array}{ll} 0 & ,m=n=0\\ {\left(\frac{1}{n}\left(\underset{\pi \in \Pi_{n}}{\text{min}}\sum_{i=1}^{m}d^{\left(c\right)}{\left(x_{i},\;y_{\pi (i)}\right)}^{p}+c^{p}(n-m)\right)\right)}^{1/p} & ,m\leq n\\ {\bar{d}}_{p}^{\left(q\right)}\;(Y,\;X) & ,m>n \end{array}$$

where Π*_n_* is the set of permutations on {1,⋯*n*}, *d*^(*c*)^(*x*,*y*) = min(*c*,*d*(*x*,*y*)), *p* is the order that penalizes error of individual element estimates, *c* is the cut-off parameter that penalizes error of cardinality estimate. We chose *p* = 2 and *c* = 30(*pixel*) in our simulation. Note that the chosen value of cut-off parameter *c* is significantly larger than the typical measurement noise but significantly smaller than the maximal distance between targets, thus maintaining a balance between the cardinality and localization components of the OSPA error [22].

### 4.2. Numerical Results

#### 4.2.1. Filtering Results for Multi-Target Tracking

**Figure 2.**
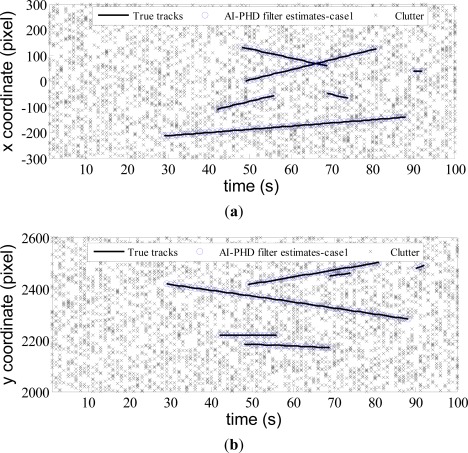
Filter estimates with known SNR.

**Figure 3.**
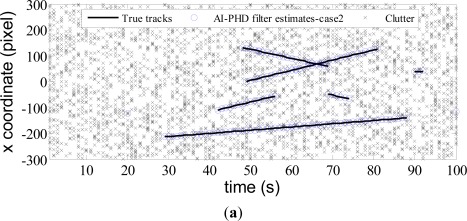

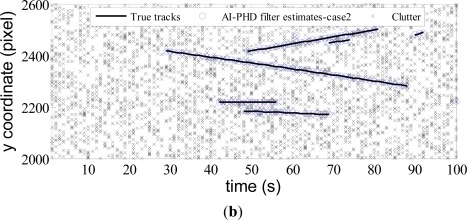
Filter estimates with unknown SNR.

The effectiveness of our AI-PHD filter for multi-target tracking in image plane of optical sensor is verified through simulation. We assume a moderately cluttered scenario that the probabilities of false alarm, $p_{\mathrm{FA}}^{\tau }=1\times 10^{-4}$, which means the clutter density, *λ* = 1 × 10^−4^ *pixel*^−2^. The SNRs of all targets are set as *d* = 6 and the probability of detection $p_{D}^{\tau }(d)\approx 0.99$ (see Table 1). For the unknown SNR case, the SNR region is set as [2,10] and the probability of detection is replaced by $p_{D}^{\tau }$ which can be computed by Equation (12). Other parameters for the filter are given as in Section 4.1. The true trajectories and filter estimates are shown in *x* and *y* coordinates of image plane *versus* time for AI-PHD filter with known and unknown target SNRs in Figures 2 and 3 respectively (denoted as case1 and case2 accordingly).

From the estimates of AI-PHD filter shown in Figures 2 and 3, we see both for the known and unknown SNR case, the filters can eliminate dense clutter in the scenario with time-varying number of targets. All targets are detected immediately after birth and tracked accurately, which highlights the track initialization, maintenance and termination capabilities of our algorithm. The results also demonstrate superior performance of the algorithm in targets number and target states estimation. We evaluate this performance by Monte Carlo simulation next.

#### 4.2.2. Monte Carlo Results and Analysis

Average OSPA and computation time cost per frame are used to evaluate the performance and computational complexity of our AI-PHD filter. To prove the improvements of the proposed method, we have benchmarked the results against a generic PHD filter which does not use the amplitude information. To make the assessment as fair as possible, the probability of detection $p_{D}^{\tau }$ for filters without amplitude information was chosen to be the same as AI-PHD filter of the known SNR case, as in Table 1. 50 Monte Carlo runs were carried out for each combination of $p_{\mathrm{FA}}^{\tau }$ and *d* on computer (Intel quad core processor 2.66 GHz, 32-bit operating system, 4 Gbytes RAM) using Matlab (R2009b). Average OSPA for AI-PHD filter of known SNR and unknown SNR case and generic PHD filter are given in Table 2 where the results are divided by ‘/’ accordingly.

**Figure 4.**
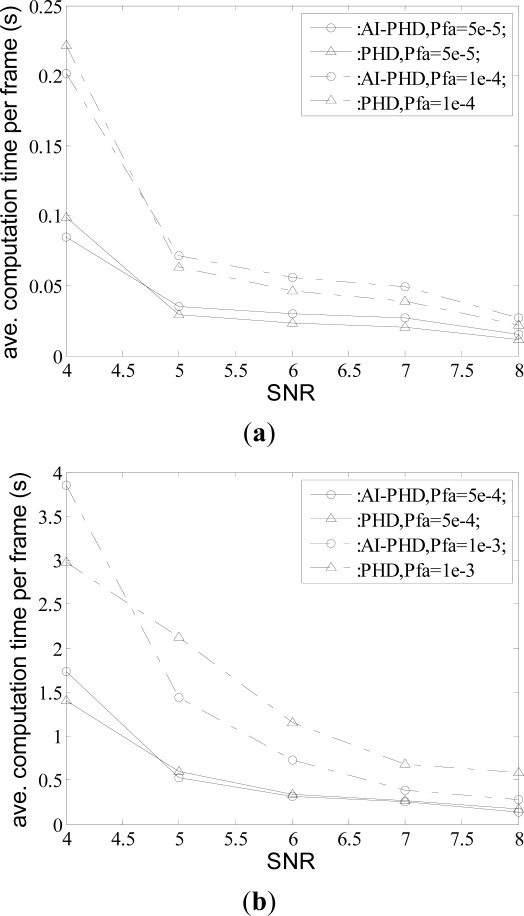
Average computation time per frame for different algorithms with different SNRs *d* and probabilities of false alarm $p_{\mathrm{FA}}^{\tau }$.

From Table 2 we see that for all combinations of $p_{\mathrm{FA}}^{\tau }$ and *d* given(corresponding to different probabilities of detection), our AI-PHD filter both with known and unknown SNR gives better performance than the generic PHD filter. This improvement is enhanced as $p_{\mathrm{FA}}^{\tau }$ or *d* increases. In the case of $p_{\mathrm{FA}}^{\tau }=1\times 10^{-3}$ and *d* = 8 where the method using the amplitude information works best, our AI-PHD filter achieves 15.94 and 12.91 lower average OSPA (*pixel*) for known and unknown SNR, respectively. This improvement in performance is mainly due to two reasons: firstly, as *d* increases, the false alarm distribution will poorly represent the target counterpart and there is a big distinction in the target and false alarm distributions; secondly, as $p_{\mathrm{FA}}^{\tau }$ increases, having more measurements aids the method using the amplitude, since we discard less useful information. Table 2 also shows that the performance of generic PHD filter without amplitude decreases rapidly as $p_{\mathrm{FA}}^{\tau }$ increases since there are more measurements from false alarms which by no means could be identified from those from targets. In contrast, we see no deterioration in the performance of AI-PHD filter in this case. For the known SNR case especially, the performance increases consistently as $p_{\mathrm{FA}}^{\tau }$ increases, which means our method works even better in a scenario with dense clutters. The comparison of computational complexity between AI-PHD filter and generic PHD filter without amplitude information is shown in average computation time per frame *versus* target SNR for different $p_{\mathrm{FA}}^{\tau }$ in Figure 4. Since we can achieve similar complexity for unknown SNR case with that of known SNR by computing Equation (27) with some fast algorithms, only the result for the known SNR case is given.

**Figure 5.**
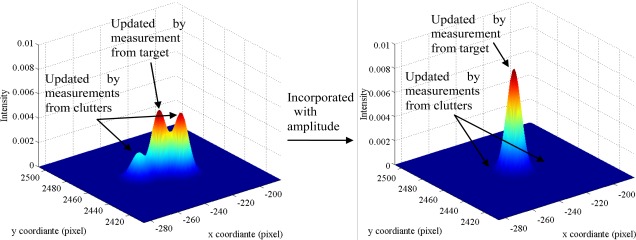
Intensity functions for generic PHD filter (**left**) and AI-PHD filter (**right**).

We see that for different given $p_{\mathrm{FA}}^{\tau }$ and *d*, the results from two filters are close with the maximum difference being no more than 1 s. Figure 4(a) shows that in the scenario with low clutter density, AI-PHD filter has only a minor increase in average computation time over the generic PHD counterpart. Furthermore, AI-PHD filter performs an even low value in high clutter density scenario which is shown in Figure 4(b). In the case of $p_{\mathrm{FA}}^{\tau }=1\times 10^{-3}$, *d* = 8 where this reduction is most obvious, the average computation time of AI-PHD filter is reduced by 53.7% over the generic PHD counterpart, which means the AI-PHD filter has even lower computational complexity than the PHD filter without amplitude information in scenarios with dense clutter and high SNR. The primary reason for this trend is that in these scenarios, the computation time cost is mainly decided by the multi-target state extraction step given the same number of targets and measurements. Incorporated with amplitude information, the update for the AI-PHD filter (see Equation (33)) gives heavier weights to the Gaussian items updated by the measurements from targets, thus updating the PHD with comparatively higher intensity near the real target positions and at the same time, suppressing the intensity of PHD near clutter positions (see Figure 5). Therefore, the updated Gaussian items can be prune and merged quickly and accurately.

## 5. Conclusions

In this paper, we have proposed a FISST-based method using signal amplitude information in a PHD filter for multi-target tracking applications in the image plane of optical sensors. We extend the measurement model to include the signal amplitude of observations and then incorporate this information into a PHD recursion in the form of an amplitude likelihood ratio. Based on the assumption that the amplitudes of the measurements from true targets are stronger than those from clutter, we show our method can significantly improve the performance over the one without amplitude information. Furthermore, simulation results also demonstrate that our method has much lower computational complexity in the scenario with high SNR and dense clutter, which makes sense for its practical implementation. Future work will involve improving the tracking performance for the targets with much lower SNR.

## Figures and Tables

**Table 1.** $p_{D}^{\tau }(d)$ under different SNR and $p_{\mathrm{FA}}^{\tau }$ combinations.

	***d***				
$p_{\mathrm{FA}}^{\tau }$	**4**	**5**	**6**	**7**	**8**
5 × 10^−5^	0.5436	0.6864	0.9825	0.9991	1.0000
1 × 10^−4^	0.6106	0.8999	0.9887	0.9995	1.0000
5 × 10^−4^	0.7610	0.9563	0.9966	0.9999	1.0000
1 × 10^−3^	0.8185	0.9719	0.9982	1.0000	1.0000

**Table 2.** Average OSPA (pixel) for different algorithms.

***d***						
$p_{\mathrm{FA}}^{\tau }$	*τ*	**4**	**5**	**6**	**7**	**8**
5 × 10^−5^	3.8906	17.42/18.88/19.00	8.17/8.230/9.65	2.93/3.71/6.08	1.57/3.53/5.78	0.97/2.69/5.27
1 × 10^−4^	3.7190	17.82/16.13/19.19	7.37/6.44/11.01	2.83/3.14/7.97	1.43/3.33/8.32	1.11/3.41/7.82
5 × 10^−4^	3.2905	16.41/13.53/20.21	6.65/5.91/15.71	2.79/4.14/14.89	1.38/4.18/15.15	0.95/3.99/14.43
1 × 10^−3^	3.0902	14.82/11.83/21.42	5.92/5.85/17.81	2.26/5.54/17.37	1.11/4.38/17.09	1.00/4.03/16.94
